# Anthropometric indicators and risk of obstructive sleep apnea: Role of HDL-C as a potential mediator

**DOI:** 10.1097/MD.0000000000051275

**Published:** 2026-10-30

**Authors:** Sheng Yue Jiang, Xue Lian Du, Zi Qian Luo, Ping Ping Xu, Ya Qi Liang, Pang Pang Zhang, Fen Ping, Ping Li

**Affiliations:** a Department of Geriatric Respiratory Medicine, Hebei General Hospital, Shijiazhuang, People’s Republic of China; b The Second People’s Hospital of Xinxiang, Xinxiang, People’s Republic of China; c Clinical Medical College, Jining Medical University, Jining, People’s Republic of China.

**Keywords:** Anthropometric Indicators, High-Density Lipoprotein Cholesterol, Lipid Metabolism, Mediating Effect, Obesity, Obstructive Sleep Apnea

## Abstract

This cross-sectional study analyzed data from 9068 U.S. adults (weighted n = 170.2 million) in the 2015–2020 NHANES. It aimed to elucidate the nonlinear relationships between six anthropometric indices – Relative Fat Mass Index (RFM), Body Roundness Index (BRI), Weight-adjusted Waist Index (WWI), Body Mass Index (BMI), and waist circumference – and obstructive sleep apnea (OSA) risk. Furthermore, we pioneered an exploration of the potential mediating role of high-density lipoprotein cholesterol (HDL-C). Multivariable logistic regression, adjusted for sociodemographic, behavioral, and clinical confounders, revealed significant positive trends between all indices and OSA risk (*P* for trend < .05). Threshold effect models and smooth curve fitting identified distinct association patterns. RFM exhibited a linear dose-response relationship with OSA (*P* < .05), whereas BRI, WWI, BMI, and waist circumference demonstrated nonlinear positive correlations with identifiable inflection points (*P* < .05). Bootstrap mediation analysis (1000 replications) yielded results consistent with a significant mediating role for HDL-C, which explained 5.6 to 21.9% of the observed associations. This effect was most pronounced for WWI (13.2%). Stratified analyses indicated enhanced associations in participants aged < 60 years (interaction *P* < .05). These findings establish RFM as a linear predictor of OSA, contrasting with the nonlinear dynamics of other adiposity indices. The partial mediation by HDL-C underscores dysregulated lipid metabolism as a key pathological mechanism linking obesity to OSA, supporting the integration of anthropometric screening with lipid profiling to optimize clinical risk stratification and early intervention strategies.

## 1. Introduction

Obstructive sleep apnea (OSA) is a prevalent sleep-related breathing disorder, characterized by intermittent hypoxia, sleep fragmentation, and excessive daytime sleepiness.^[1]^ Current estimates suggest that globally, 936 million adults aged 30 to 69 years have mild-to-severe OSA, and 425 million have moderate-to-severe OSA.^[2]^ OSA can lead to various complications, including hypertension, diabetes, cardiovascular disease, dementia, and cerebrovascular disease.^[3–5]^ Consequently, there is an urgent need to develop more targeted metrics for a comprehensive assessment of OSA risk.

Obesity is widely regarded as a primary risk factor for OSA, particularly central obesity,^[6]^ which exerts a direct influence on the stability of the upper airway through mechanical compression,^[7]^ inflammatory response, and oxidative stress.^[8]^ However, traditional body measurement indicators such as body mass index (BMI) have certain limitations in assessing the risk of OSA. Specifically, BMI is unable to effectively distinguish between different distributions of body fat, including visceral fat. Consequently, novel composite obesity-related indicators, such as relative fat mass (RFM), body roundness index (BRI), and weight-adjusted waist index (WWI), have been employed in recent years to enhance our understanding of metabolic diseases. These indicators have been shown to offer an enhanced ability to reflect the distribution of abdominal fat and are closely related to metabolic health status. Current studies have demonstrated the correlation between BRI, WWI, WHR^[9–11]^ and OSA. However, the mechanism of these indicators in OSA risk assessment still needs to be further explored. RFM is an equation calculated by Woolcott et al in 2018, using data from the National Health and Nutrition Examination Survey (NHANES).^[12]^ This survey included 365 anthropometric indicators. The calculation method incorporates gender-based disparities in height and waist circumference, which is a more accurate estimate of the percentage of total body fat in adults than BMI.^[13]^ RFM has been demonstrated to serve as a reliable marker of obesity in the general population, and it has also been found to be closely related to diabetes, nonalcoholic fatty liver disease, asthma, and cardiovascular-related diseases.^[14–18]^ However, its relationship with OSA has not been found.

Furthermore, obesity-related metabolic disorders, most notably aberrant high-density lipoprotein cholesterol (HDL-C) levels, are also considered to interact with oxidative stress and inflammatory processes in OSA. Animal experiments have demonstrated that intermittent hypoxia can down-regulate the expression of Peroxisome Proliferator-Activated Receptor Alpha (PPARα) in the liver,^[19]^ thereby reducing the synthesis of HDL-C.^[20]^ Furthermore, Fadaei et al measured HDL cholesterol efflux capacity (CEC) in OSA patients and found that HDL function was impaired in these patients and was associated with increased markers related to inflammatory levels, such as high-sensitivity C-reactive protein (hsCRP) and apolipoprotein B (apo B).^[21]^

This study integrated RFM and other multidimensional anthropometric indicators and was based on big data from the NHANES. For the first time, the present study jointly analyzed the strength and threshold characteristics of the association between OSA and six indicators, including RFM, BRI, WWI, BMI, and waist circumference (WAIST). In addition, the study investigated and quantified the potential mediating role of HDL-C in these associations. The findings of this study are expected to enhance the comprehension of the metabolic pathological mechanisms of OSA and to furnish novel targets for individualized intervention strategies. This study not only enhances the efficacy of risk assessment in OSA cases, but also fosters the development of personalized intervention strategies that are tailored to individual metabolism and morphology.

## 2. Material and methods

### 2.1. Study population

The NHANES is a comprehensive program designed to assess the health and nutritional status of Americans. Each survey cycle encompasses demographic data, physical measurements, laboratory tests, and dietary information. Detailed methods and documentation are accessible on the NHANES website. The present study utilized data from the 2015–2016 and 2017–2020 NHANES cycles, encompassing a total of 25,531 individuals, with 9068 subjects remaining after various exclusions. Participants with the following characteristics were excluded from the study: missing data on OSA-related questionnaires; age < 20 years; lacking anthropometric indicators such as RFM, WWI, BRI, BMI, and waist circumference; and missing data on HDL-C and other covariates. This resulted in a final sample of 9068 participants (Fig. 1).

**Figure 1.**
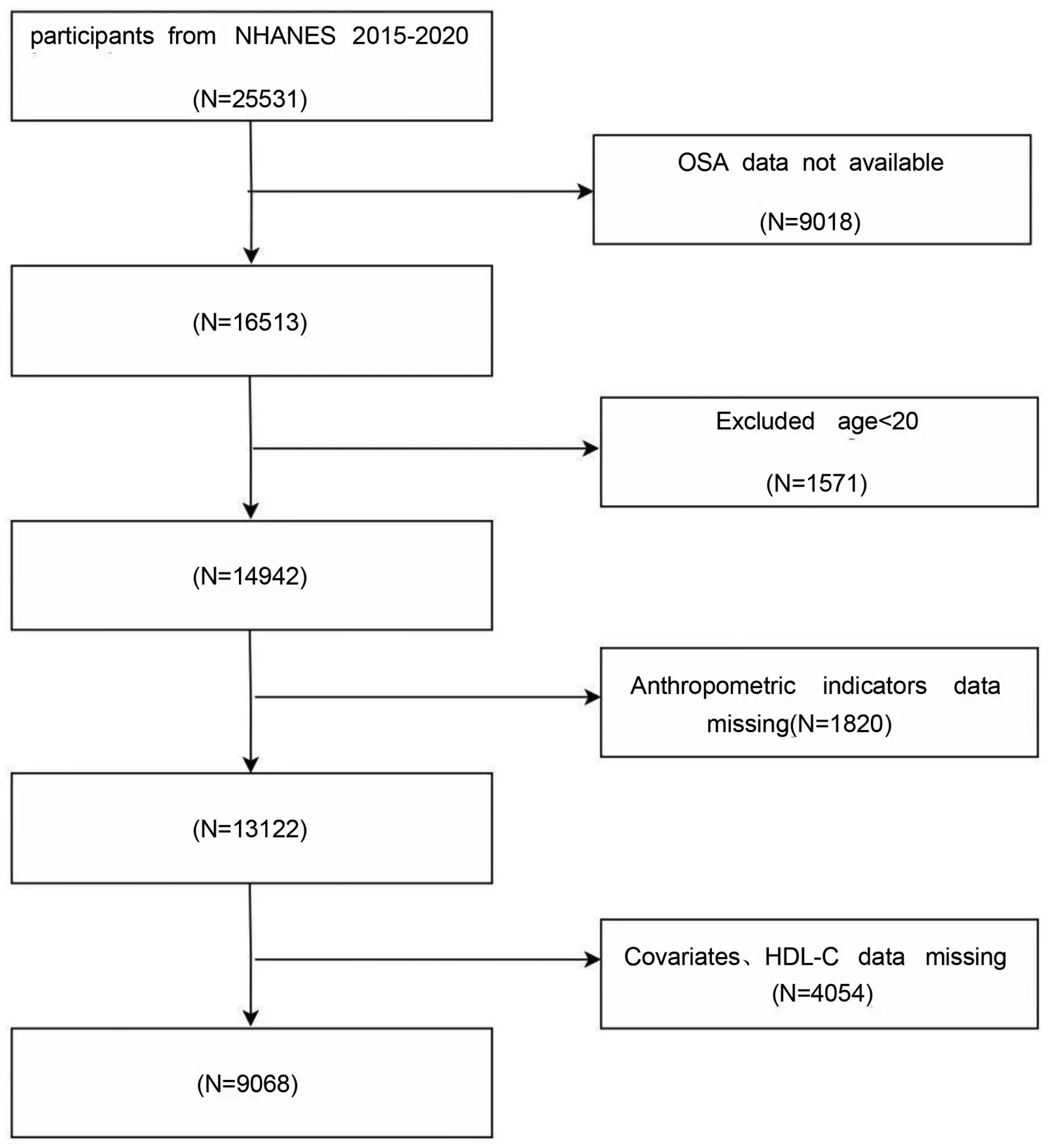
Flowchart of participant screening. HDL-C = high-density lipoprotein-cholesterol, NHANES = National Health and Nutrition Examination Survey, OSA = obstructive sleep apnea.

### 2.2. Anthropometric indicators

Anthropometric data, including height, weight, and waist circumference, were collected by trained health technicians stationed at mobile examination centers, following standardized protocols. NHANES health technicians undergo comprehensive training before their assignments, and their performance is regularly reviewed and evaluated by expert reviewers to ensure protocol compliance and data accuracy.

The anthropometric indicators were calculated using the following formulas, where sex was coded as female = 1 and male = 0:

WAIST = waist circumference (cm)

BMI = weight (kg)/ height (m)^2^

WWI = waist circumference (cm)/ √weight (kg)

BRI = 364.2 − 365.5 × (1 − [waist circumference (cm)/ 2π]^2^/ [0.5 × height (m)] ^2^)

RFM = 64 − (20 × height (cm)/ waist circumference (cm)) + (12 × sex)

### 2.3. Definition of OSA

Participants were considered to have OSA if they met one or more of the following criteria:^[22–24]^ Self-reported snoring more than 3 nights per week; Despite sleeping ≥ 7 hours at night, they reported feeling excessively sleepy 16 to 30 times per month, regardless of weekdays or weekends; Self-reported episodes of apnea, gasping, or snorting more than 3 nights per week. It should be noted that this definition relies on self-reported symptoms, which, while practical for large-scale screening, may be subject to recall bias.

### 2.4. Covariates

The covariates in this study included demographic, socioeconomic, anthropometric, lifestyle, and health-related factors. Specifically, the selected covariates were age, sex, race, education level, marital status, ratio of family income to the poverty line (PIR), HDL-C, alcohol consumption, smoking status, history of hypertension, diabetes, and cardiovascular disease. Race was categorized into Mexican American, non-Hispanic White, non-Hispanic Black, other Hispanic, and other races. Educational attainment was divided into 3 levels: less than high school, high school diploma, and more than high school. Marital status was defined as either “married/cohabiting” or “living alone.” Questionnaires were used to collect information on smoking, alcohol consumption, and medical history of hypertension, diabetes, and cardiovascular disease. The term drinking 4/5 or more per day was defined as an indicator of drinking status. Smoking status was determined based on whether participants had smoked 100 or more cigarettes in their lifetime. Histories of hypertension, diabetes, and cardiovascular disease were based on self-reports of diagnoses by a physician or other health professional. All study variables are accessible via the NHANES website at www.cdc.gov/nchs/nhanes/.

### 2.5. Statistical analysis

This study adhered to the CDC guidelines, employing NHANES sample weights for weighted analyses. The specific analytical procedures were as follows: assessment of demographic characteristics: for continuous variables, weighted means and 95% confidence intervals (CI) were calculated, with *P*-values determined via weighted linear regression. Categorical variables were described using weighted percentages and 95% CI, with *P*-values from weighted chi-square tests. To explore the association between anthropometric indicators and OSA prevalence, we used a weighted logistic regression model, including unadjusted, minimally adjusted, and fully adjusted models. Model 1 was unadjusted. Model 2 adjusted for age, sex, and race. Model 3 further adjusted for education, marital status, PIR, alcohol consumption, smoking, hypertension, diabetes, and cardiovascular disease, based on Model 2. We performed weighted multiple logistic regression to elucidate the association between anthropometric indicators and OSA, treating the metrics as both continuous and categorical variables (quartiles). Trend estimation was done by considering the quartiles as a continuous variable. Generalized Additive Models (GAMs) were used for smoothing spline fitting to identify potential nonlinear relationships. Subsequently, we employed weighted multivariate logistic regression analysis to examine the association between HDL-C and the prevalence of OSA. In addition, in order to evaluate the possible mediating role of HDL-C on the association between anthropometric indicators and OSA, the Bootstrap method (1000 repeated sampling) was used to test the association suggesting mediation of HDL-C, and the formula was calculated: proportion of mediating effect = (indirect effect/total effect) × 100%. Finally, subgroup analyses and interaction tests were performed on potential confounders detailed in the baseline tables. Statistical analyses were performed with the use of EmpowerStats (version 4.2) and R software (version 4.2.0). The threshold for statistical significance was set at *P* < .05.

## 3. Results

### 3.1. Population baseline characteristics

The study included 9068 participants, representing a weighted sample of 170,186,708. Among them, there were 4633 OSA patients, representing a weighted sample of 86,120,316 people. There were 4435 non-OSA patients, representing a weighted sample of 84,066,392. Overall, males accounted for 49.89% (95% CI = 48.60, 51.17), and the average age was 47.88 years (95% CI = 46.95, 48.81). The OSA group was found to be older (49.55 vs 46.24 years), had a higher proportion of males (55.31% vs 44.60%), and had significantly higher obesity-related indicators: BMI, WAIST, BRI, RFM, and WWI all demonstrated *P* < .001, but the HDL-C levels were lower (*P* < .001). The prevalence of hypertension, diabetes, and cardiovascular disease was significantly higher in the OSA group (*P* < .05), and the prevalence of smoking (51.72% vs 41.55%) and drinking (18.61% vs 11.89%) was higher (*P* < .001). In terms of socioeconomic status, their family income index (PIR 3.12 vs 3.25) and proportion of higher education (62.15% vs 68.77%) were lower (*P* < .001). However, more were married/cohabiting (68.97% vs 59.74%). Table 1 provides the combined baseline characteristics of the remaining populations.

**Table 1:** Baseline characteristics of participants OSA and Non-OSA, NHANES, 2017–2020.

Characteristic	Overall N = 170186708	Non-OSA N = 84066392	OSA N = 86120316	*P*
Continuous variables: survey-weighted mean (95% CI)				
PIR	3.19 (3.09,3.29)	3.25 (3.14,3.36)	3.12 (3.00,3.23)	.016
Age (years)	47.88 (46.95,48.81)	46.24 (45.33,47.16)	49.55 (48.51,50.60)	<.001
BMI (kg/m2)	29.72 (29.41,30.03)	28.03 (27.62,28.44)	31.45 (31.15,31.75)	<.001
WAIST (cm)	101.05 (100.25,101.85)	96.55 (95.56,97.55)	105.65 (104.87,106.44)	<.001
HDL-C (mmol/L)	1.41 (1.39,1.43)	1.48 (1.45,1.51)	1.34 (1.32,1.36)	<.001
BRI	5.62 (5.51,5.73)	5.05 (4.91,5.18)	6.21 (6.10,6.33)	<.001
RFM	35.75 (35.41,36.09)	34.94 (34.49,35.39)	36.58 (36.20,36.96)	<.001
WWI	11.03 (10.98,11.07)	10.88 (10.83,10.92)	11.18 (11.13,11.22)	<.001
Categorical variables: survey-weighted percentage (95% CI)				
Sex				<.001
Male	49.89 (48.60,51.17)	44.60 (42.48,46.74)	55.31 (53.34,57.26)	
Female	50.11 (48.83,51.40)	55.40 (53.26,57.52)	44.69 (42.74,46.66)	
Race				.048
Mexican American	7.92 (6.14,10.16)	7.18 (5.45,9.39)	8.68 (6.69,11.19)	
Other Hispanic	6.25 (5.01,7.78)	5.86 (4.67,7.33)	6.66 (5.22,8.45)	
Non-Hispanic White	67.93 (63.70,71.89)	68.83 (64.52,72.85)	67.01 (62.59,71.15)	
Non-Hispanic Black	9.89 (7.90,12.32)	9.96 (7.77,12.68)	9.82 (7.91,12.14)	
Other races	8.00 (6.80,9.40)	8.17 (6.73,9.88)	7.83 (6.60,9.26)	
Educational level				<.001
Below high school	9.82 (8.60,11.20)	8.32 (6.98,9.90)	11.36 (9.97,12.91)	
High school or general education	24.68 (22.58,26.91)	22.91 (20.10,25.98)	26.50 (24.21,28.92)	
High school or above	65.50 (62.54,68.34)	68.77 (65.03,72.27)	62.15 (59.19,65.02)	
Alcohol drinking				<.001
Yes	15.21 (13.90,16.62)	11.89 (10.43,13.52)	18.61 (17.02,20.32)	
No	84.79 (83.38,86.10)	88.11 (86.48,89.57)	81.39 (79.68,82.98)	
Hypertension				<.001
Yes	32.05 (30.26,33.90)	26.03 (23.96,28.20)	38.23 (35.75,40.77)	
No	67.95 (66.10,69.74)	73.97 (71.80,76.04)	61.77 (59.23,64.25)	
Diabetes				<.001
Yes	10.60 (9.70,11.57)	8.07 (7.20,9.03)	13.19 (11.87,14.63)	
No	87.13 (86.08,88.12)	89.96 (88.84,90.98)	84.23 (82.56,85.77)	
borderline	2.27 (1.87,2.75)	1.97 (1.43,2.71)	2.58 (2.00,3.33)	
Congestive heart-failure				.51
Yes	2.23 (1.87,2.64)	2.10 (1.61,2.74)	2.35 (1.89,2.91)	
No	97.77 (97.36,98.13)	97.90 (97.26,98.39)	97.65 (97.09,98.11)	
Coronary heart disease				.08
Yes	3.82 (2.97,4.89)	3.33 (2.47,4.47)	4.32 (3.24,5.73)	
No	96.18 (95.11,97.03)	96.67 (95.53,97.53)	95.68 (94.27,96.76)	
Angina pectoris				.0095
Yes	1.84 (1.51,2.24)	1.37 (1.03,1.82)	2.31 (1.75,3.04)	
No	98.16 (97.76,98.49)	98.63 (98.18,98.97)	97.69 (96.96,98.25)	
Myocardial infarction				.0007
Yes	3.29 (2.65,4.09)	2.52 (1.80,3.53)	4.08 (3.38,4.92)	
No	96.71 (95.91,97.35)	97.48 (96.47,98.20)	95.92 (95.08,96.62)	
Stroke				.01
Yes	2.93 (2.52,3.39)	2.37 (1.97,2.86)	3.49 (2.79,4.37)	
No	97.07 (96.61,97.48)	97.63 (97.14,98.03)	96.51 (95.63,97.21)	
CVD				<.001
Yes	8.77 (7.76,9.90)	7.21 (6.00,8.63)	10.37 (9.25,11.61)	
No	91.23 (90.10,92.24)	92.79 (91.37,94.00)	89.63 (88.39,90.75)	
Smoke				<.001
Yes	46.58 (44.52,48.65)	41.55 (38.64,44.52)	51.72 (49.44,54.00)	
No	53.42 (51.35,55.48)	58.45 (55.48,61.36)	48.28 (46.00,50.56)	
Marriage				<.001
Married/cohabiting	64.30 (61.89,66.64)	59.74 (56.31,63.08)	68.97 (66.77,71.09)	
solitude	35.70 (33.36,38.11)	40.26 (36.92,43.69)	31.03 (28.91,33.23)	

BMI = body mass index, BRI = body roundness index, CI = confidence interval, CVD = cardiovascular disease, HDL-C = high-density lipoprotein-cholesterol, NHANES = National Health and Nutrition Examination Survey, OSA = obstructive sleep apnea, PIR = ratio of family income to Poverty, RFM = relative fat mass, WAIST = waist circumference, WWI = weight-adjusted waist index.

### 3.2. Anthropometric indicators and obstructive sleep apnea

In three multivariable regression analyses, we further explored the associations between anthropometric indicators and OSA (Table 2). The results showed that, in both unadjusted and adjusted models, anthropometric indicators were significantly positively correlated with OSA risk (all *P* for trend < .05). In the fully adjusted model 3, each 1-cm increase in WAIST was associated with a 3% increase in OSA risk (OR = 1.03, 95% CI = 1.02, 1.03, *P* < .001), with an OR of 3.60 (95% CI = 2.84, 4.57, *P* < .001) for those in the highest quartile, and a significant dose-response relationship (*P* for trend < .001). For BMI, each 1 kg/m^2^ increase was linked to a 7% increase in risk (OR = 1.07, 95% CI = 1.06, 1.08, *P* < .001); Quartile 4 (OR = 3.37, 95% CI = 2.66, 4.27, *P* < .001), with a significant trend (*P* < .001). BRI showed a 22% increase in risk per unit increase (OR = 1.22, 95% CI = 1.18, 1.26, *P* < .001), and Quartile 4 (OR = 3.48, 95% CI = 2.74, 4.42, *P* < .001), with P for trend < .001. RFM was associated with a 9% increase in risk per unit increase (OR = 1.09, 95% CI = 1.08, 1.11, *P* < .001), and Quartile 4 was markedly elevated (OR = 5.08, 95% CI = 3.76, 6.87, *P* < .001), with a significant trend (*P* for trend < .001). WWI showed a 53% increase in risk per unit increase (OR = 1.53, 95% CI = 1.37, 1.71, *P* < .001), and Quartile 4 (OR = 2.17, 95% CI = 1.78, 2.66, *P* < .001), with *P* for trend < .001.

**Table 2:** Association between Anthropometric Indicators and OSA in patients of NHANES.

	Model 1	Model 2	Model 3
	OR (95%CI) *P*	OR (95%CI) *P*	OR (95%CI) *P*
BRI			
Continuous	1.25 (1.21, 1.28) < .001	1.26 (1.22, 1.30) < .001	1.22 (1.18, 1.26) < .001
Quartiles			
Quartile1	Ref.	Ref.	Ref.
Quartile2	2.01 (1.67, 2.43) < .001	1.87 (1.54, 2.26) < .001	1.70 (1.39, 2.08) ≤ .001
Quartile3	2.88 (2.38, 3.49) < .001	2.71 (2.21, 3.33) < .001	2.31 (1.85, 2.88) < .001
Quartile4	4.02 (3.28, 4.93) < .001	4.23 (3.39, 5.26) < .001	3.48 (2.74, 4.42) < .001
*P* for trend	<.001	<.001	<.001
RFM			
Continuous	1.02 (1.02, 1.03) < .001	1.11 (1.09, 1.12) < .001	1.09 (1.08, 1.11) < .001
Quartiles			
Quartile1	Ref.	Ref.	Ref.
Quartile2	1.62 (1.42, 1.86) < .001	2.10 (1.78, 2.49) < .001	1.83 (1.53, 2.19) < .001
Quartile3	1.05 (0.87, 1.27).6039	3.01 (2.26, 4.01) < .001	2.44 (1.82, 3.27) < .001
Quartile4	1.76 (1.52, 2.05) < .001	7.09 (5.42, 9.27) < .001	5.08 (3.76, 6.87) < .001
*P* for trend	<.001	<.001	<.001
WWI			
Continuous	1.58 (1.48, 1.70) < .001	1.74 (1.58, 1.91) < .001	1.53 (1.37, 1.71) < .001
Quartiles			
Quartile1	Ref.	Ref.	Ref.
Quartile2	1.62 (1.35, 1.95) < .001	1.63 (1.35, 1.97) < .001	1.38 (1.14, 1.67).0046
Quartile3	2.10 (1.68, 2.62) < .0001	2.19 (1.73, 2.76) < .0001	1.73 (1.35, 2.21).0005
Quartile4	2.52 (2.21, 2.87) < .0001	2.88 (2.43, 3.41) < .001	2.17 (1.78, 2.66) < .001
*P* for trend	<.001	<.001	<.001
WAIST			
Continuous	1.04 (1.03, 1.04) < .001	1.03 (1.03, 1.04) < 0.001	1.03 (1.02, 1.03) < 0.001
Quartiles			
Quartile1	Ref.	Ref.	Ref.
Quartile2	2.05 (1.70, 2.49) < .001	1.85 (1.52, 2.25) < .001	1.70 (1.37, 2.10).0002
Quartile3	2.70 (2.28, 3.21) < .001	2.39 (2.02, 2.81) < .001	2.06 (1.71, 2.48) < .001
Quartile4	4.83 (3.95, 5.91) < .001	4.40 (3.58, 5.41) < .001	3.60 (2.84, 4.57) < .001
*P* for trend	<.001	<.001	<.001
BMI			
Continuous	1.08 (1.07, 1.09) < .001	1.08 (1.07, 1.09) < .001	1.07 (1.06, 1.08) < .001
Quartiles			
Quartile1	Ref.	Ref.	Ref.
Quartile2	1.76 (1.47, 2.11) < .001	1.55 (1.30, 1.86) < .001	1.46 (1.21, 1.78).0014
Quartile3	2.81 (2.32, 3.40) < .001	2.52 (2.08, 3.04) < .001	2.26 (1.85, 2.77) < .001
Quartile4	4.04 (3.26, 5.01) < .001	4.02 (3.25, 4.96) < .001	3.37 (2.66, 4.27) < .001
*P* for trend	<.001	<.001	<.001

Model 1: Unadjusted.

Model 2: Adjusted for sex, age, race.

Model 3: Adjusted for the variables in Model 2 together with marriage educational level, PIR, Alcohol drinking, hypertension, diabetes, congestive heart-failure, coronary heart disease, angina pectoris, myocardial infarction, stroke, smoke.

BMI = body mass index, BRI = body roundness index, CI = confidence interval, HDL-C = high-density lipoprotein-cholesterol, NHANES = National Health and Nutrition Examination Survey, OR = odds ratio, OSA = obstructive sleep apnea, PIR = ratio of family income to Poverty, RFM = relative fat mass, WAIST = waist circumference, WWI = weight-adjusted waist index.

### 3.3. Curve fitting and threshold effect analysis

To further visualize the associations between anthropometric indicators and OSA, smooth curve fitting based on Model 3 was conducted, adjusting for all covariates (Fig. 2). Threshold effect analyses were also performed to clarify these relationships (Table 3). The results of the study demonstrate that RFM showed a positive linear relationship with OSA (*P* for likelihood ratio test = .197). In contrast, BRI, WWI, BMI, and WAIST demonstrated nonlinear positive associations (*P* for likelihood ratio test < .05). A distinct inflection point for WAIST was identified at 123.2 cm. Below this value, each 1-cm increase in WAIST was linked to a 3% increase in OSA risk (OR = 1.03, 95% CI = 1.03, 1.03, *P* < .001), but risk plateaued above this threshold. For WWI, the inflection point was at 11.7; below this, each unit increase raised risk by 59% (OR = 1.59, *P* < .001), with a weaker association above it. BRI showed increasing risk below the inflection point of 9.98 (OR = 1.24, *P* < .001) but no significant association beyond it (OR = 0.96, *P* = .2486). Similar patterns were observed for BMI (inflection point: 40.6 kg/m^2^), indicating differing impacts on OSA risk depending on whether values were below or above these thresholds. These inflection points offer quantifiable critical values for OSA risk stratification.

**Table 3:** The threshold effect of Anthropometric Indicators on OSA analyzed by a two-stage logistic regression model.

Exposure	BRI	RFM	WWI	BMI	WAIST
	Adjusted OR(95%) *P*	Adjusted OR(95%) *P*	Adjusted OR(95%) *P*	Adjusted OR(95%) *P*	Adjusted OR(95%) *P*
One-line effect	1.19 (1.17, 1.22) < .001	1.09 (1.08, 1.10) < .001	1.46 (1.36, 1.55) < .001	1.06 (1.06, 1.07) < .001	1.03 (1.02, 1.03) < .001
Inflection point(K)	9.98	—	11.7	40.6	123.2
< K slope	1.24 (1.21, 1.28) < .001	1.09 (1.08, 1.10) < .001	1.59 (1.46, 1.73) < .001	1.08 (1.07, 1.09) < .001	1.03 (1.03, 1.03) < .001
> K slope	0.96 (0.90, 1.03) .2486	1.03 (0.95, 1.12) .5026	1.13 (0.96, 1.34) .1466	1.00 (0.98, 1.02) .8114	1.01 (0.99, 1.02) .3357
*P* for likelihood ratio test	<.001	.197	.002	<.001	<.001

Adjusted for sex, age, race, marriage, educational level, PIR, HDL-C, Alcohol drinking, hypertension, diabetes, congestive heart-failure, coronary heart disease, angina pectoris, myocardial infarction, stroke, smoke.

BMI = body mass index, BRI = body roundness index, CI = confidence interval, HDL-C = high-density lipoprotein-cholesterol, OR = odds ratio, OSA = obstructive sleep apnea, PIR = ratio of family income to Poverty, RFM = relative fat mass, WAIST = waist circumference, WWI = weight-adjusted waist index.

**Figure 2.**
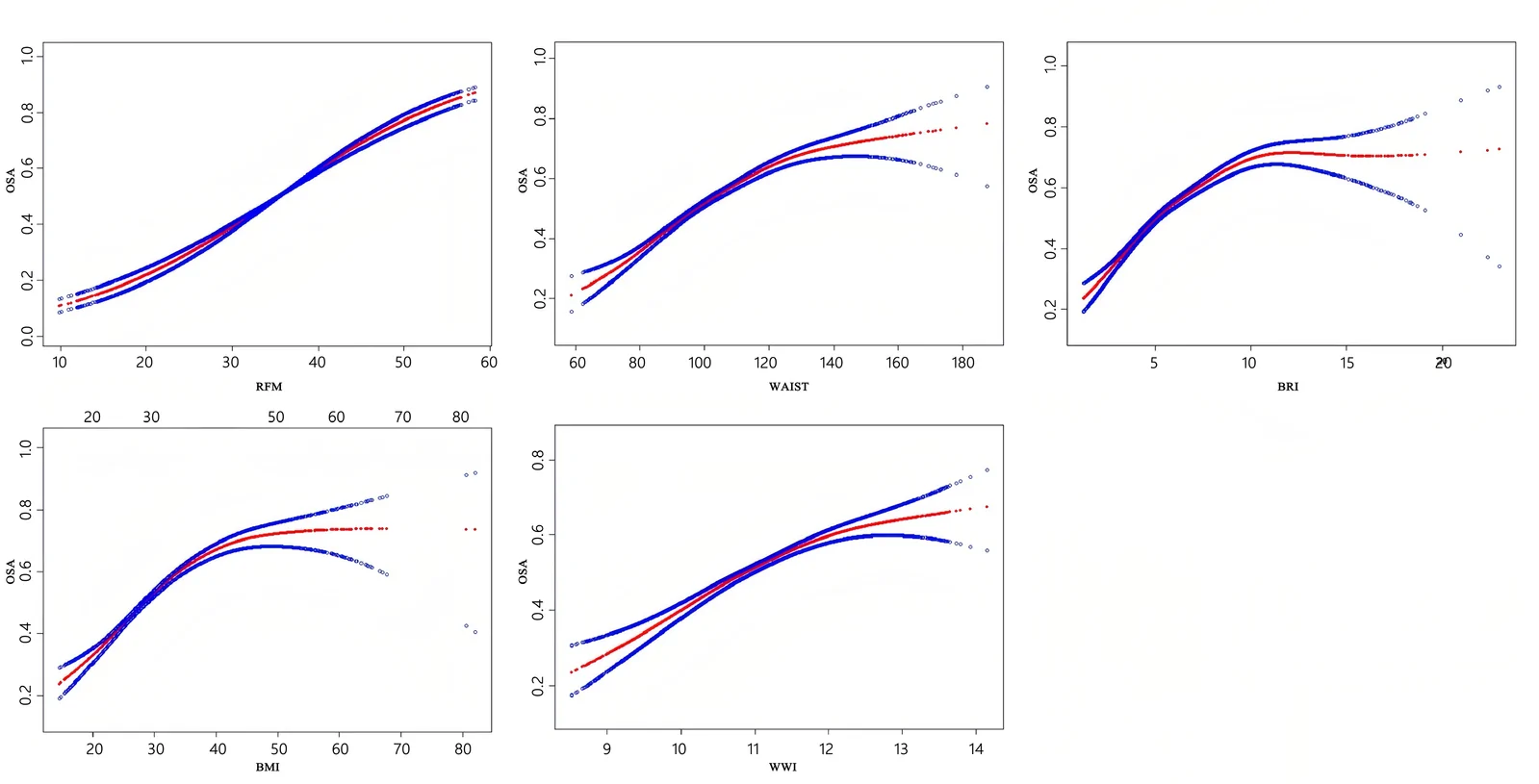
Smoothed curve-fitting plots of the relationship between BRI, RFM, BMI, WAIST, and WWI with OSA. Figure legends: The solid red line illustrates the smooth curve fit between the variables, while the blue bands depict the 95% confidence interval derived from the fit. BMI = body mass index, BRI = body roundness index, OSA = obstructive sleep apnea, RFM = relative fat mass, WAIST = waist circumference, WWI = weight-adjusted waist index.

### 3.4. High-density lipoprotein cholesterol and obstructive sleep apnea

Multiple logistic regression analysis revealed a significant inverse association between HDL-C levels and OSA risk. In the fully adjusted model (Model 3), analysis of HDL-C as a continuous variable showed that each 1 mmol/L increase in HDL-C was associated with a 49% reduction in OSA risk (OR = 0.51, 95% CI = 0.45, 0.58, *P* < .001). In quartile analysis, participants in the highest quartile (Quartile 4) had a 49% lower risk of OSA compared to those in the lowest quartile (Quartile 1) (OR = 0.51, 95% CI = 0.43, 0.60, *P* < .001), with a significant dose-response relationship (*P* for trend < .001) (Table 4).

**Table 4:** Association between HDL-C and OSA in patients of NHANES.

characteristic	Model 1	Model 2	Model 3
	OR (95%CI) *P*	OR (95%CI) *P*	OR (95%CI) *P*
HDL-C (mmol/L) (Continuous)	0.45 (0.39, 0.51) < .001	0.46 (0.41, 0.53) < .001	0.51 (0.45, 0.58) < .001
HDL-C (quartiles)			
Quartile1	Ref.	Ref.	Ref.
Quartile2	0.78 (0.65, 0.93).0088	0.79 (0.66, 0.95).0177	0.85 (0.71, 1.02).1035
Quartile3	0.53 (0.45, 0.63) < .001	0.56 (0.46, 0.67) < .001	0.62 (0.51, 0.74) ≤ .001
Quartile4	0.42 (0.36, 0.49) < .001	0.45 (0.38, 0.53) < .001	0.51 (0.43, 0.60) < .001
*P* for trend	<.001	<.001	<0.001

Model 1: Unadjusted.

Model 2: Adjusted for sex, age, race.

Model 3: Adjusted for the variables in Model 2 together with marriage educational level, PIR, Alcohol drinking, hypertension, diabetes, congestive heart-failure, coronary heart disease, angina pectoris, myocardial infarction, stroke, smoke.

CI = confidence interval, HDL-C = high-density lipoprotein-cholesterol, NHANES = National Health and Nutrition Examination Survey, OR = odds ratio, OSA = obstructive sleep apnea, PIR = ratio of family income to Poverty.

### 3.5. Association suggesting mediation of HDL-C on anthropometric indicators and OSA

We further examined the association suggesting mediation of HDL-C in the associations between various anthropometric indicators and OSA risk using mediation analysis (Fig. 3, Table 5). The results showed that HDL-C exhibited the highest association suggesting mediation in the association between WHR and OSA, with a mediating proportion of 21.90%. Additionally, WWI showed a mediating proportion of 13.17%. Among other anthropometric indicators, the association suggesting mediation proportions of HDL-C were 7.27% for BRI, 6.63% for BMI, 6.57% for WAIST, and 5.58% for RFM. Despite variations in the association suggesting mediation of HDL-C across different metrics, its role in moderating the relationship between anthropometric indicators and OSA risk remains significant.

**Table 5:** Estimated proportion of the association between Anthropometric Indicators and OSA mediated by HDL-C.

	Mediation effect (95% CI), *P*			
	Total effect	Indirect effect	Direct effect	Mediation
Exposure				
WWI	0.116558 (0.100028, 0.132851) <.001	0.015356(0.011074, 0.019775) <.001	0.101305 (0.084230, 0.118054) <.001	13.17%
BRI	0.133877 (0.119248, 0.149161) < .001	0.009737 (0.005458, 0.014103) <.001	0.124146 (0.108777, 0.139127) < .001	7.27%
BMI	0.132972 (0.118670, 0.147388) <.001	0.008823 (0.004610, 0.013132) <.001	0.124157 (0.109242, 0.138958) <.001	6.63%
WAIST	0.147080 (0.131837, 0.162400) <.001	0.009658 (0.004761, 0.014540) <.001	0.137467 (0.121068, 0.153215) <.001	6.57%
RFM	0.255105 (0.234547, 0.274917) <.001	0.014229 (0.005520, 0.023341) .0040	0.241271 (0.218449, 0.262638) <.001	5.58%

Adjusted for sex, age, race, marriage, educational level, PIR, Alcohol drinking, hypertension, diabetes, congestive heart-failure, coronary heart disease, angina pectoris, myocardial infarction, stroke and smoke.

BMI = body mass index, BRI = body roundness index, CI = confidence interval, HDL-C = high-density lipoprotein-cholesterol, OR = odds ratio, OSA = obstructive sleep apnea, PIR = ratio of family income to Poverty, RFM = relative fat mass, WAIST = waist circumference, WWI = weight-adjusted waist index.

**Figure 3.**
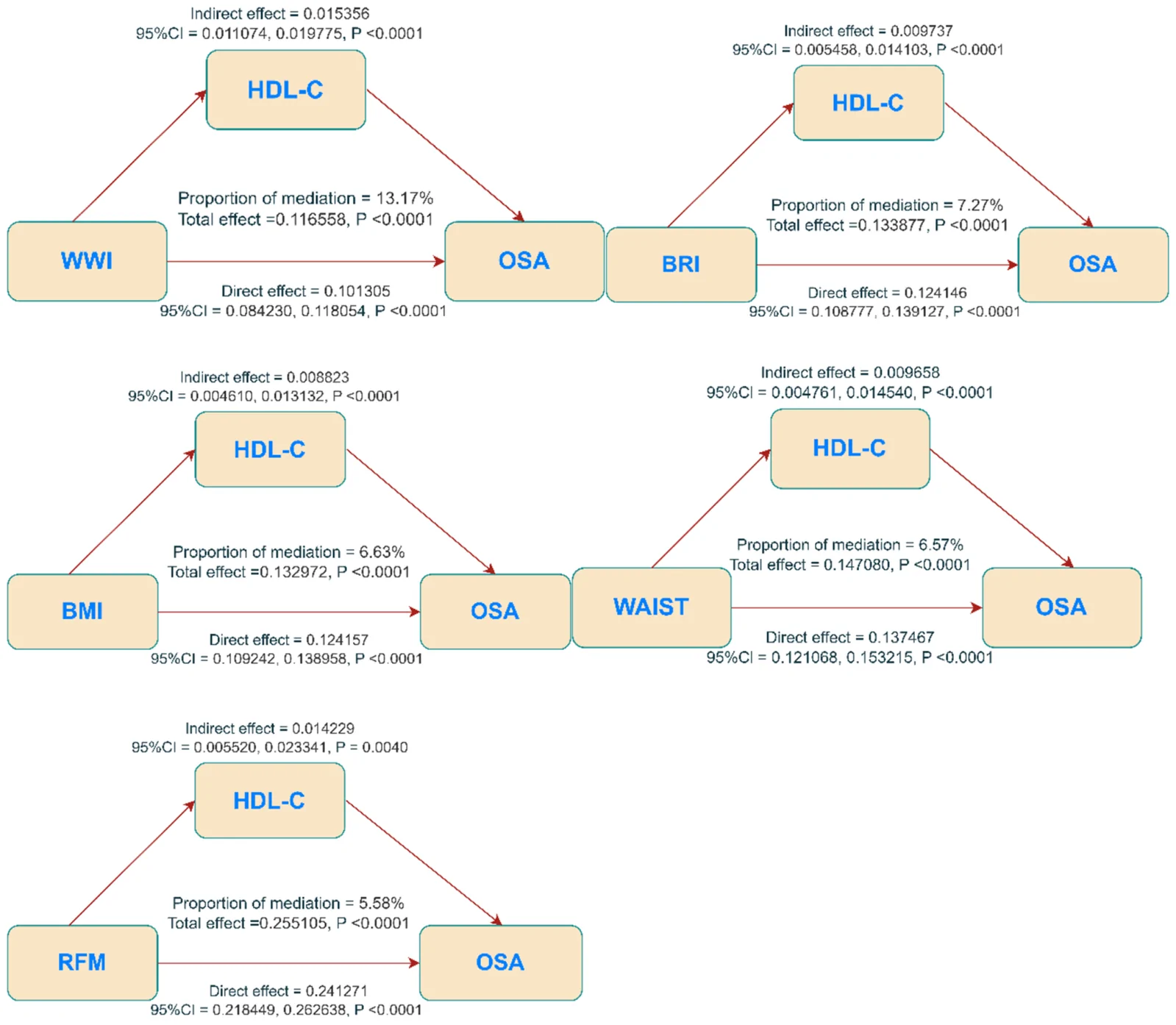
Mediation analysis of HDL-C between anthropometric indicators and OSA. Adjusted for sex, age, race, marriage, educational level, PIR, alcohol drinking, hypertension, diabetes, congestive heart failure, coronary heart disease, angina pectoris, myocardial infarction, stroke, and smoke. BMI = body mass index, BRI = body roundness index, CI = confidence interval, OSA = obstructive sleep apnea, RFM = relative fat mass, WAIST = waist circumference, WWI = weight-adjusted waist index, HDL-C = high-density lipoprotein-cholesterol, PIR = ratio of family income to poverty.

### 3.6. Subgroup analysis

To evaluate the stability of the associations between anthropometric indicators and OSA risk across different populations, we conducted subgroup analyses. The results showed a consistent positive correlation between these metrics and OSA risk across all subgroups. Age emerged as a significant moderator, with notable differences in association strength across age groups (interaction *P* < .05). Notably, individuals younger than 60 years exhibited a distinct pattern, suggesting that central obesity may have a more pronounced driving effect on OSA in younger populations (Figs. S1–S5, Supplemental Digital Content 1).

## 4. Discussion

In this cross-sectional study, we analyzed NHANES data from 2015 to 2020 in order to investigate the association between anthropometric indicators and OSA. The findings of this study demonstrate a consistent correlation between RFM and OSA risk within this demographic, exhibiting a linear increase. However, the relationship between BRI, WWI, BMI, WAIST, and OSA risk demonstrated a more complex nonlinear pattern. Furthermore, it was also found for the first time that HDL-C exhibited a significant association suggesting mediation in the association between anthropometric indicators and OSA. Our study expands upon previous research focused on measures like WWI and BRI,^[9,10]^ which have shown nonlinear associations with OSA risk. In contrast, we found that the Relative Fat Mass (RFM) index exhibits a strong linear relationship with OSA. This suggests that RFM may more accurately reflect visceral adiposity relevant to OSA pathophysiology. Our results position RFM as a potentially superior and more predictable metric for OSA risk stratification in clinical practice.

Obesity is a recognized core risk factor for OSA, and the association between obesity and OSA is largely attributed to the accumulation of abdominal fat. This visceral fat has been demonstrated to exert a greater influence on the upper airway than peripheral fat. In a study involving 122 obese patients, abdominal fat distribution was measured by bioelectrical impedance analysis (BIA), and OSA was diagnosed by polysomnography. The results demonstrated that visceral fat area exhibited a significant correlation with OSA and functioned as an independent risk factor for OSA.^[6]^ These findings underscore the pivotal function of visceral adipose tissue in the pathophysiology of OSA. The present study incorporated six anthropometric indicators: RFM, BRI, WWI, BMI, and WAIST. BMI reflects overall weight distribution, while BRI, WWI, and WAIST indicate abdominal fat distribution. Our findings are consistent with those of previous studies demonstrating an association between obesity-related indicators and OSA. Furthermore, a novel measure of fat distribution, RFM, was introduced to assess the percentage of total body fat by differences in waist circumference, height, and sex. Research has demonstrated a significant correlation between RFM and various health outcomes, including diabetes, heart disease, and related mortality, as well as all-cause mortality. Xiao et al conducted a study to evaluate the relationship between RFM and the risk of diabetes in a Japanese population. The findings indicated the presence of a positive nonlinear relationship and threshold effect between RFM and diabetes risk, and that keeping RFM at a low level may help to reduce diabetes risk.^[25]^ Utilizing data from the NHANES (n = 46,535), the study by Woolcott et al determined that RFM exhibited a significant correlation with diabetes-related mortality, heart disease mortality, and all-cause mortality, and emerged as a more effective predictor of mortality in comparison to BMI and waist circumference. Research findings indicated a stronger correlation between RFM and diabetes mortality. Furthermore, all measures demonstrated a significant association with heart disease and all-cause mortality. The results of the NHANES III validation process demonstrated consistency. It is likely that RFM is a more effective indicator for assessing the risk of obesity-related mortality.^[26]^ Our study found that, in Model 3, the risk increase in the RFM Q2-Q3 group was flat (Q2 OR = 1.83, Q3 OR = 2.44), while the risk in the Q4 group increased rapidly (OR = 5.08). It is suggested that clinical intervention should focus on individuals with very high body fat percentage.

In the mediation analysis model, RFM, BRI, WWI, BMI, and WAIST have been shown to increase the risk of OSA. However, HDL levels seem to amplify the effect of these indicators on OSA risk, exhibiting a significant association consistent with mediation. Furthermore, this provides a rationale for combined interventions, such as GLP-1 receptor agonists, to improve visceral fat distribution.^[27]^ The study by Popadic et al demonstrated a significant correlation between HDL and the severity of OSA, with patients exhibiting lower HDL levels demonstrating higher apnea-hypopnea index (AHI) values. This finding aligns with the results of our own investigation.^[28]^ However, our study has some limitations, and confounding by lipid-lowering agents cannot be ruled out. Therefore, the mediation results should be viewed as exploratory, and future studies with detailed medication use data are needed to confirm our findings.

HDL-C may play a dual regulatory role in the relationship between obesity and OSA. On the one hand, obesity can lead to significant changes in HDL metabolism, composition, and subclass distribution. Impaired HDL function may be associated with low-grade inflammation and metabolic disorders associated with obesity. In their analysis of 316 participants, Tang et al observed a shift in the distribution of HDL subclasses, accompanied by a substantial increase in lymphocytes and inflammatory markers, including hs-CRP and IL-6, in individuals exhibiting metabolically unhealthy/obese states. Levels of HDL2b were found to be inversely correlated with inflammatory markers, whereas high levels of HDL3 and low levels of HDL2b were associated with adverse metabolic status. It has been hypothesized that HDL2b exerts an anti-inflammatory effect by modulating lymphocyte activation. This finding suggests that it could be a viable therapeutic target for metabolic diseases.^[29]^ On the other hand, through the reverse cholesterol transport process, HDL-C transports cholesterol from peripheral tissues to the liver for excretion, thereby reducing visceral fat accumulation and consequently reducing compression of the upper airway.^[30]^ The antioxidant and anti-inflammatory functions of HDL-C may inhibit the activation of the NF-κB pathway, thereby reducing upper airway inflammation and the severity of OSA.^[31]^ This dual mechanism of action provides a novel perspective on the relationship between obesity and OSA.^[32]^

The research results of the subgroup analysis also demonstrated that adults under the age of 65 exhibited more significant associations in the relationship between anthropometric indicators and OSA risk. This phenomenon is associated with age-related changes in metabolic rate and body fat distribution, with younger age groups potentially demonstrating a heightened susceptibility to central obesity. The diversity of physiological factors associated with increasing age may result in the effects of anthropometric indicators becoming less pronounced in older age groups. Furthermore, the elderly population is frequently characterized by the presence of chronic diseases and other uncontrolled confounders, which have the potential to obscure the association with OSA risk.

Overall, this study deepens the understanding of the relationship between anthropometric indicators and OSA risk, with particular emphasis on the association suggesting mediation of HDL-C. It is recommended that future studies consider longitudinal designs to fully elucidate the mechanisms underlying the effects of these indicators on OSA development and explore the potential of improving HDL-C levels in reducing OSA risk. These findings provide an important theoretical basis for individualized intervention and risk assessment, which helps to develop more effective intervention strategies and improve the prognosis of OSA patients. Despite the valuable insights provided by this study, there are several limitations that must be acknowledged. Firstly, due to the cross-sectional study design, it was not possible to definitively establish a causal relationship between anthropometric indicators and OSA. Secondly, the diagnosis of OSA is typically reliant upon polysomnography (PSG), a process that necessitates the implementation and analysis of data using specialized instruments by trained professionals. In contrast, although questionnaires can be used in a variety of settings for the initial screening of patients with OSA, it must be noted that they have the potential to introduce recall bias and inaccuracy compared to objective recordings of sleep activity.^[33]^ For instance, some patients with moderate-to-severe OSA may not report typical symptoms and could be missed by the questionnaire, potentially leading to an underestimation of OSA prevalence.^[34]^ Consequently, the reported associations between anthropometric indices and OSA may represent conservative estimates of the true relationship strengths. The present study was constrained by a paucity of research data, which precluded the observation of any differences between OSA of varying severity. Furthermore, the study was primarily based on samples from the United States, which may limit the generalizability of the results to other regions.

## 5. Conclusion

This study unveils a multifaceted interplay between OSA risk and anthropometric indicators. RFM demonstrated a dose-dependent positive linear association, while BRI, WWI, BMI, and WC exhibited a significant nonlinear positive correlation pattern. This discrepancy may be attributable to the heterogeneity of visceral fat distribution. This suggests that central obesity can magnify the pathogenic effect of OSA through lipid metabolism disorders. This finding has two implications for clinical practice: firstly, that differentiated screening thresholds must be established according to the type of indicator; and secondly, that lipid regulation should be included in the core target of OSA prevention. It is recommended that future studies be conducted to verify the dynamic association suggesting mediation of HDL-C through the utilization of longitudinal cohorts. Furthermore, it is advised that such studies explore the regulatory effect of lipid-lowering treatment on OSA risk.

## Acknowledgments

We acknowledge the NHANES database for facilitating access to their valuable data platforms. We also extend our appreciation to the contributors who have diligently uploaded their significant datasets, which have been instrumental in our research endeavors.

## Author contributions

**Conceptualization:** Sheng Yue Jiang.

**Formal analysis:** Sheng Yue Jiang, Ya Qi Liang, Pang Pang Zhang.

**Methodology:** Sheng Yue Jiang, Xue Lian Du.

**Writing – original draft:** Sheng Yue Jiang, Xue Lian Du, Zi Qian Luo, Ping Ping Xu.

**Data curation:** Xue Lian Du, Ya Qi Liang.

**Investigation:** Zi Qian Luo.

**Software:** Zi Qian Luo.

**Visualization:** Zi Qian Luo.

**Writing – review & editing:** Ping Ping Xu, Fen Ping, Ping Li.

**Validation:** Pang Pang Zhang.

**Project administration:** Fen Ping, Ping Li.

**Supervision:** Fen Ping, Ping Li.

**Funding acquisition:** Ping Li.

**Resources:** Ping Li.










